# Exophtalmie pulsatile révélant une agénésie sphéno-orbitaire dans la maladie de Von-Recklinghausen

**DOI:** 10.11604/pamj.2016.25.104.9006

**Published:** 2016-10-21

**Authors:** Imane Ait Belaid, Safouane Khairallah, Soumaya Alj, Mariem Ouali Idrissi, Najat Cherif Idrissi El Ganouni

**Affiliations:** 1 Department of Radiology, Mohammed VI Teaching Hospital, Cadi Ayyad University, Marrakesh, Morocco

**Keywords:** Exophtalmie pulsatile, maladie de Von-Recklinghausen, agénésie sphéno-orbitaire, Pulsatile exophthalmos, Von Recklinghausen's disease, spheno-orbital agenesis

## Abstract

Les manifestations ophtalmologiques au cours de la maladie de Von Recklinghausen sont rares. Seulement quelques cas ont été rapportés dans la littérature internationale. Nous rapportons un cas d'exophtalmie pulsatile révélatrice d'une agénésie sphéno-orbitaire au cours de la maladie de Von Recklinghausen.

## Abstract (en)

Ophthalmological manifestations of Von Recklinghausen disease are rare. Only a few cases have been reported in the international literature. We here report the case of a patient with pulsatile exophthalmos revealing spheno-orbital agenesis associated with Von Recklinghausen's disease.

## Introduction

L'exophtalmie pulsatile par atteinte sphénoïdale est très rare, toujours décrite dans le cadre de la neurofibromatose de type I et s'associe souvent à un névrome plexiforme de la paupière. Elle constitue une urgence fonctionnelle qui peut être révélatrice de la pathologie. Le diagnostic repose sur l'imagerie, notamment le scanner qui permet de faire le bilan des anomalies osseuses et l'IRM qui permet d'affiner le diagnostic et d'éliminer les autres diagnostics différentiels notamment la fistule carotido-caverneuse. Nous rapportons le cas d'une agénésie sphéno-orbitaire responsable d'une exophtalmie pulsatile unilatérale chez un homme atteint de la maladie de Recklinghausen.

## Patient et observation

**Figure 1:**
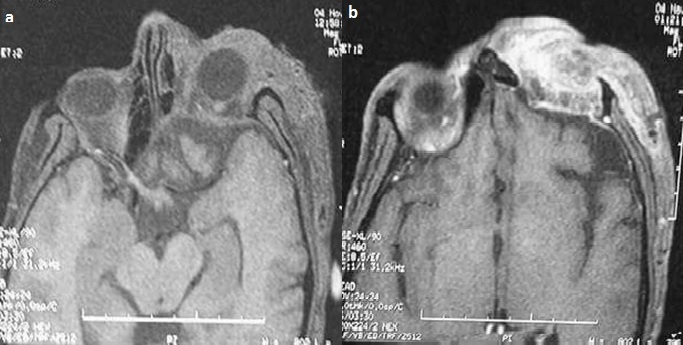
Coupe axiale en séquence T1 sans (a) et après injection du Gadolinium (b) masse tumorale centrée sur la paupière supérieur gauche, en hypo signal T1, rehaussée de façon intense après injection de produit de contraste, englobant le globe oculaire et s’étend du cantus interne vers l’externe infiltrant la graisse extra-conique

**Figure 2:**
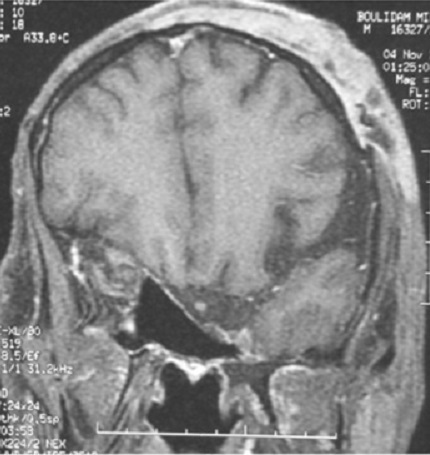
Coupe coronale en séquence T1 avec injection: éffondrement de la partie postéro-supérieur du toit de l’orbite ainsi que la partie gauche du sinus sphénoïdale avec ptose du parenchyme frontal et les espaces sous arachnoïdiens

**Figure 3:**
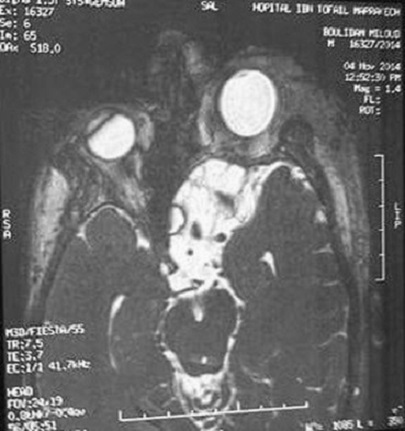
Coupe axiale en séquence fiesta, déhiscence de la grande aile du sphénoïde gauche avec hernie du parenchyme temporal et du LCR dans l’orbite

Il s'agit d'un patient âgé de 40 ans, connu porteur d'une neurofibromatose type 1, hospitalisé au service de chirurgie maxillo-faciale pour prise en charge d'un névrome plexiforme de la paupière supérieure gauche. L'examen clinique a mis en évidence une exophtalmie pulsatile gauche. Une fistule carotido-caverneuse a été suspectée. Une IRM orbito-cérébrale a été réalisée. Des séquences pondérées en T1, T2, FIESTA et T1 en saturation de graisse avant et après injection de produit de contraste ainsi qu'une angio-IRM artérielle ont été réalisées. Elles ont objectivées une masse tumorale centrée sur la paupière supérieure gauche, en hyposignal T1, hypersignal T2, rehaussée de façon intense après injection du produit de contraste. Elle englobe tout le globe oculaire et s'étend du cantus interne vers l'externe en infiltrant la graisse extra-conique, le muscle droit supérieur, droit externe et grand oblique (Figure 1). Il s'y associe un effondrement de la partie postéro-supérieur du toit de l'orbite ainsi que la partie gauche du sinus sphénoïdale avec ptose du parenchyme frontal et les espaces sous arachnoïdiens (Figure 2), une déhiscence de la grande aile du sphénoïde gauche avec hernie du parenchyme temporal et du LCR dans l'orbite (Figure 3), les sinus caverneux étaient sans anomalies, et il n'y a pas eu de signes en faveur de la fistule carotido-caverneuse.

## Discussion

L'exophtalmie pulsatile est une protrusion globulaire résultant souvent d'une pathologie vasculaire type fistule carotido-caverneuse, rarement retrouvée dans le cadre de neurofibromatose type 1 ou maladie de Von-Recklinghausen [1]. La maladie de Von-Recklinghausen est la plus fréquente des phacomatoses, caractérisée par la présence des neurofibromes qui sont des tumeurs bénignes et hétérogènes de la gaine des nerfs périphériques disséminées dans différents territoires (peau, tissus cellulaires sous cutanés, profond). Les lésions céphaliques représentent 3 à 7% des atteintes au cours de la neurofibromatose de Von Recklinghausen [2]. La région orbito-palpébrale est le siège de prédilection de l'atteinte céphalique au cours de la neurofibromatose de type I, caractérisée par des neurofibromes type névrome plexiforme de la paupière supérieure qui sont pathognomoniques de la NF1. Il est associée à d´autres manifestations orbito-faciales dont les plus fréquentes sont représentées par l'hypertrophie de l'hémiface homolatérale et la dysplasie sphéno-orbitaire [3]. Cette dernière réalise un amincissement ou une déhiscence totale des éléments constitutifs du sphénoïde à l'origine d'une déformation facio-orbitaire avec élargissement de l'orbite, de la fissure orbitaire supérieure et de la fosse temporale, responsable d'une communication entre le parenchyme fronto-temporal et le fond de l'orbite réalisant une hernie méningo-encéphalique. Cliniquement, il associe une plagiocéphalie avec une exophtalmie pulsatile qui reste rare, due à l'expansion cérebro-méningée dans la partie supérieure de l'orbite [4]. La dysplasie de la grande aile sphénoïdale est associée à un névrome plexiforme dans 50 à 100% des cas, ce qui explique l'aggravation progressive de la dysplasie avec l'âge [5]. Sur le plan radiologique, la radiographie standard constitue une aide précieuse pour la détection des anomalies osseuses au cours de l'agénésie sphéno-orbitaire, objectivant l'absence de la grande aile du sphénoïde, avec agrandissement de l'orbite et élévation de la petite aile du sphénoïde et du toit de l'orbite [6]. Ces lésions osseuses sont mieux analysées à la TDM qui représente le principal moyen d'imagerie pour la dysplasie sphéno-orbitaire, objectivant une dysplasie partielle ou totale ou un simple amincissement de la grande aile du sphénoïde [7]. L'IRM permet de mieux étudier les tumeurs nerveuses notamment le neurofibrome plexiforme, qui parait en hyposignal relatif par rapport au muscle en T1, en hypersignal T2 si la lésion est volumineuse, un hypo signal central réalisant un aspect en cocarde caractéristique, le rehaussement est variable : central, diffus, périphérique, ou en cible [8]. Le traitement chirurgicale de la dysplasie sphéno-orbitaire reste réservé pour les cas compliqués d'une hernie meningo-encéphalique ayant entrainé une exophtalmie importante ou une baisse de l'acuité visuelle par compression du nerf optique. Cependant, Morax et Coll [9] proposent une approche neurochirurgicale avec réduction de la hernie méningo-encéphalique et reconstruction de l'apex orbitaire à l'aide de greffes osseuses.

## Conclusion

La dysgénésie orbito-sphénoïdale est une cause peu connue d'exophtalmie. Elle doit être évoquée dans le cadre de la neurofibromatose dès lors que l'exophtalmie est pulsatile. Inversement, sa constatation doit conduire à un examen clinique et radiologique à la recherche de signes pouvant faire évoquer une neurofibromatose de Recklinghausen.
